# Design, Characterization, and Incorporation of the Alkaline Aluminosilicate Binder in Temperature-Insulating Composites

**DOI:** 10.3390/ma17030664

**Published:** 2024-01-29

**Authors:** Pavlo Kryvenko, Igor Rudenko, Oleksandr Konstantynovskyi, Oleksandr Gelevera

**Affiliations:** Scientific Research Institute for Binders and Materials, Kyiv National University of Construction and Architecture, Povitroflotskyi Prospect 31, 03037 Kyiv, Ukraine; rudenko.ii@knuba.edu.ua (I.R.); konstantynovskyi.op@knuba.edu.ua (O.K.); gelevera.og@knuba.edu.ua (O.G.)

**Keywords:** alkaline aluminosilicate binder, composition design, binding, composite, curing conditions, durability, fire-proofness, heat insulation, structure formation, zeolite-like reaction products

## Abstract

This paper covers the design of binder formulations and technology for low-energy building materials based on alkaline aluminosilicate binders developed for special uses. The microstructure of the binders was investigated using scanning electron and atomic force microscopy examination techniques. The identification of phase compositions was performed by means of X-ray diffraction. The degree of binding of the alkali metal ions within the binder was determined with the help of chemical analysis of the pore fluid. Structure formation depending upon binder mix design and curing conditions was also studied. Some examples of the manufacture and application of binder-based glues and adhesives, including those developed for heat insulation and fire prevention, are discussed. The advantages of binder-based temperature-insulating composite materials compared with traditionally used materials are highlighted.

## 1. Introduction

In view of the severe consequences of today’s military actions, terrorism, and natural disasters, the demand for the reliable protection of critical military and civil infrastructure is increasingly growing. The requirements for such protective materials are the following: the use of easy-to-produce mineral binders as a binding agent, environmental friendliness, low energy consumption, and excellent performance properties under various severe conditions, e.g., high temperatures and fire. Aside from the above problems, nowadays, it is critical to reduce energy consumption through the use of highly efficient insulating composite materials.

One method of synthesizing alkaline aluminosilicate hydrates, the analogs of natural zeolites, using a mixture of natural (clays) and artificial aluminosilicates with alkali metal compounds under various curing conditions was established by Victor Glukhovsky [1]. This method is based on the modeling (simulating) of natural processes during the formation of alkaline zeolite-like aluminosilicates at different temperatures. An important conclusion was drawn: an increase in temperature promotes a smooth dehydration process and subsequent recrystallization of the hydration products with the formation of anhydrous aluminosilicates of alkali metals, i.e., Li, Na, K, Rb, and Cs [2]. Alkali metal compounds not only act as activators of hardening but are also responsible for the formation of the main structural elements of alkali-activated materials. These reaction products, being analogous to natural zeolites of the Na_2_O(K_2_O)∙Al_2_O_3_∙(2…4)SiO_2_∙nH_2_O type, have been discovered in ancient concretes (e.g., in Ancient Greece, Ancient Rome, Egypt, Syria) [3]. The durability of ancient concretes and the similarity of their structure to that of alkali-activated cement concretes allowed us to infer their high durability. The excellent performance properties of alkali-activated materials, such as their high strength and heat, corrosion, and freeze–thaw resistance [4,5,6,7], have been confirmed by over 65 years’ experience of their use. The composition of zeolite-like products is determined by structural features of aluminosilicate components (precursor), alongside their composition, the applied curing conditions, the rate of chemical reactions, and the binder design [8,9,10,11,12]. The most appropriate materials for achieving the mentioned goals are natural clays in a dehydrated state, volcanic rocks, and industrial by-products like fuel ashes, slags of various origin, and red mud [9,13,14,15,16,17,18,19].

Alkali-activated materials can be divided into alkaline cements and geopolymers (or geocements) [20]. Depending on the precursor type, the following reaction products can be formed, CaO-Al_2_O_3_-SiO_2_-H_2_O, R_2_O-CaO-Al_2_O_3_-SiO_2_-H_2_O, and R_2_O-Al_2_O_3_-SiO_2_-H_2_O, along with R–Na, K, and Li [21]. In terms of composition, the binders can be divided into high-calcium, intermediate-calcium (hybrid), and low-calcium binders [22].

Several studies have been conducted on the production of composite materials based on zeolite-like matrices of the low-calcium Na_2_O(K_2_O)-Al_2_O_3_-SiO_2_-H_2_O system, i.e., alkaline aluminosilicate binders [23,24,25,26,27].

An analysis of this research showed that the use of zeolite-like binder systems for the production of building materials for general and special applications presents a solution to the problems associated with durability, energy-saving ability, and environmental protection, where traditional binder systems fail [13,23].

Due to the polymerization of the aluminosilicate constituent, reaction products that are analogs to natural minerals (zeolites and feldspathoids) are formed. Considering their compositional make-up, zeolites may be referred to as inorganic polymers, which, on the one hand, are analogs to organic ones (in terms of such properties as elasticity, corrosion resistance, and adhesion to various materials) and, on the other hand, due to their mineral nature, are ecologically pure and heat- and fire-resistant [9,13]. As the theoretical premise for the creation of alkali-activated materials with a polymeric structure, this study aimed at the synthesis of analogs to natural minerals based on clays of various structural types and applied alkali metal compounds [3,8,26]. An extensive practical experiment of the application of these binding systems in different materials for construction and protection was conducted [28,29,30,31,32,33,34,35,36,37,38]. 

The purpose of this study was to test an approach to designing alkaline aluminosilicate binders (further, the binder) and developing a manufacturing technology for various curing conditions, especially for normal-temperature curing conditions, and for various applications.

## 2. Raw Materials and Testing Techniques

A binder of the system Na_2_O(K_2_O)-Al_2_O_3_-SiO_2_-H_2_O with varying ratios of oxides (Na_2_O(K_2_O)/Al_2_O_3_ and SiO_2_/Al_2_O_3_) was used as the object of this study.

The major components of the binder used in this study are as follows:

- Metakaolin (oxide content, % by mass: CaO—0.27; SiO_2_—54.08; Al_2_O_3_—43.61; Fe_2_O_3_—0.77; MgO—0.52; K_2_O + Na_2_O—0.25; LOI—0.50) (specific surface area = 800 m^2^/kg (by Blaine)) and kaolin (oxide content, % by mass: CaO—0.80; SiO_2_—58.50; Al_2_O_3_—39.50; Fe_2_O_3_—0.50; TiO_2_—0.70; LOI—0.19) (specific surface area = 800 m^2^/kg (by Blaine)) were used as aluminosilicate components;
- Soluble sodium silicate (per CAS 1344-09-8, silicate modulus Ms = 2.8, density = 1430 Kg/m^3^) was used as an alkaline component.

In order to adjust the composition of the binder with regard to the contents of main oxides, the following additives and alkali compounds were used:

- Tripoli powder (per CAS 1317-95-9) (oxide content, % by mass: CaO—0.83; SiO_2_—85.59; Al_2_O_3_—6.20; Fe_2_O_3_—3.15; MgO—0.95; SO_3_—0.39; TiO_2_—2.03; K_2_O + Na_2_O—0.67), specific surface area = 800 m^2^/kg (by Blaine), bulk density = 2300 kg/m^3^, porosity ≥ 70%, degree of purity 98.0%;
- Solution of potassium hydroxide KOH (per CAS 1310-58-3), density= 1430 Kg/m^3^;
- Solution of sodium hydroxide NaOH (per CAS 1310-73-2), density= 1430 Kg/m^3^.

The liquid component of the binder was prepared by mixing the soluble sodium silicate with solutions of KOH and NaOH. The solid component of the binder was prepared by mixing metakaolin or kaolin with the Tripoli powder. The binder was prepared by mixing the alkaline component with the solid component in a Hobart mixer. The ratio of binder components was calculated in order to ensure the required oxide composition of the binder, (Na_2_O + K_2_O)·Al_2_O_3_·mSiO_2_⋅nH_2_O.

The compressive strength of the alkaline aluminosilicate binding agent was determined per the EN 196-1:2016 standard with the following modifications: testing on 40 × 40 × 160 mm specimens (binder: CEN sand = 1:3) and hardening at t = 20 ± 2 °C and RH= 60 ± 5% for 28 days.

The water resistance was evaluated by considering the coefficient of water resistance (K_w_) of the 40 × 40 × 40 mm specimens (binder: CEN sand = 1:1) hardened for 28 days at t = 20 ± 2 °C and RH= 60 ± 5%. After 28 days, part of the specimens was placed into the water until almost full water saturation was achieved (the changes in mass of the specimens over 24 h did not exceed 0.2%). The coefficient of water resistance is the ratio of the compressive strength of the water-saturated specimen to that of the specimen hardened for 28 days. A material can be considered water-resistant when K_w_ ≥ 0.8. 

The compressive and flexural strengths of the binder-based perlite-based materials (lightweight concrete) were determined per the EN 12390-3:2019 and EN 12390-5:2019 standards, respectively.

Adhesion was evaluated by considering the adhesion strength of the adhesive joints between the adhesive and various substrates and the characteristics of the connection of the adhesive with various materials, which were determined per the EN 24624:1992 standard.

The identification of the phase composition of the binders was achieved through X-ray diffraction (XRD), using a single-crystal X-ray diffractometer (DRON-3M, JSC “Bourevestnik”, St. Petersburg, Russia) equipped with a copper anode and nickel filter at U = 30 kV, I = 10 to 20 mA, and a range of registered angles (2θ) (8 to 60) (the rotation speed of the counter was calculated as 2 degrees per minute using the technique reported in [39]). The X-ray spectra were identified according to references [40,41,42]. 

The microstructure of the binders was examined using a scanning electron microscope (SEM) with microanalyzer (REMMA 102-02, JSC “SELMI”, Sumy, Ukraine). The settings of SEM were as follows: accelerating voltage—35 kV; SEI mode resolution—5 nm; and magnification—from 2500 to 23000. The settings of the energy-dispersive X-ray spectrometer were as follows: analyzed element range—Na; energy resolution—143 eV at MnKα; and energy range—up to 30 kV. The microstructure was also examined using an atomic force microscope (AFM) (NT–206, ALC «Microtestmachines», Gomel, Republic of Belarus) designed by the V.A. Belii Institute for Mechanics of Metal-polymer Systems of the National Academy of Sciences of The Republic of Belarus. 

The degree of binding of the ions of alkali metals in the hardening binder was studied by the application of two methods of analysis (pH measurements and chemical analysis of the pore fluid) [43]. The measurement of pH values of the pore fluid of the binder was carried out using a pH meter, PL-700AL.

## 3. Results and Discussion

### 3.1. The Influence of Basic Binder Composition and Curing Conditions on Structure Formation of the Alkaline Aluminosilicate Binder

#### 3.1.1. Influence of Binder Design

**Figure 1:**
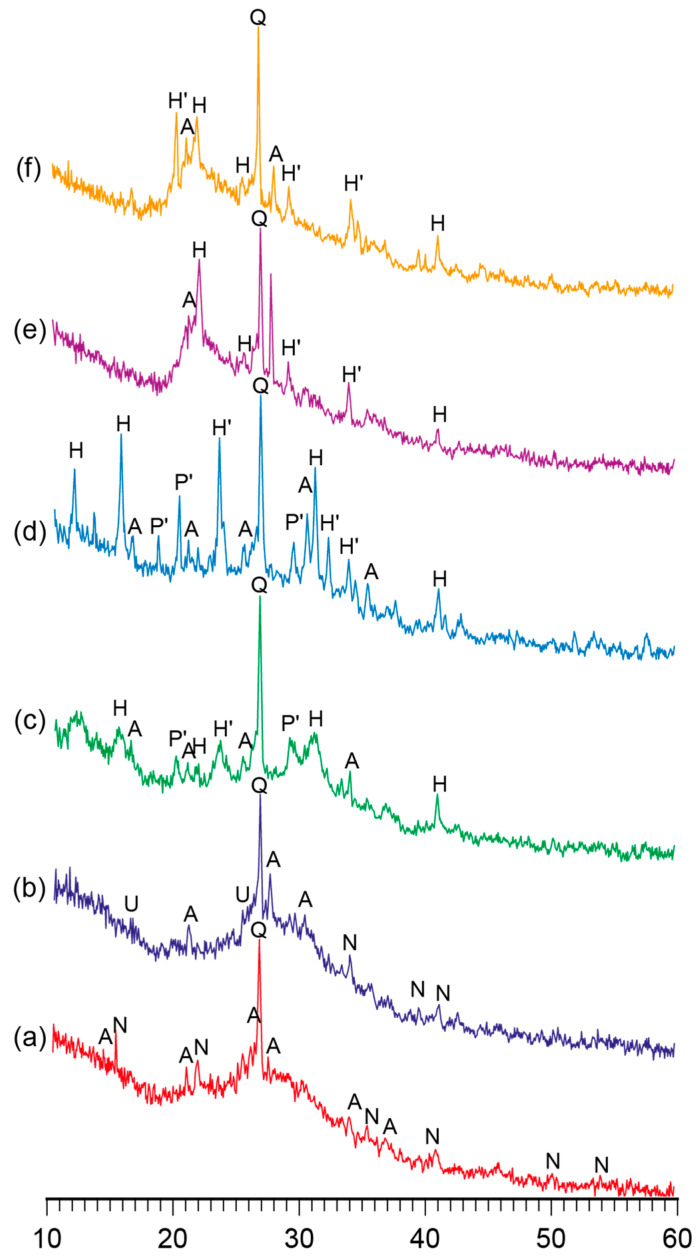
The XRD patterns of the binder with varying K_2_O/(Na_2_O+K_2_O) = 0.15 and SiO_2_/Al_2_O_3_ ratios, namely (**a**) 2, (**b**) 3, (**c**) 4, (**d**) 5, (**e**) 6, and (**f**) 7, after curing at t = 80 °C. Abbreviations: Q—quartz; N—natrolite; A—analcime; U—ussingite; P’—Na–K phillipsite; H—Na heulandite; H’—K heulandite.

**Figure 2:**
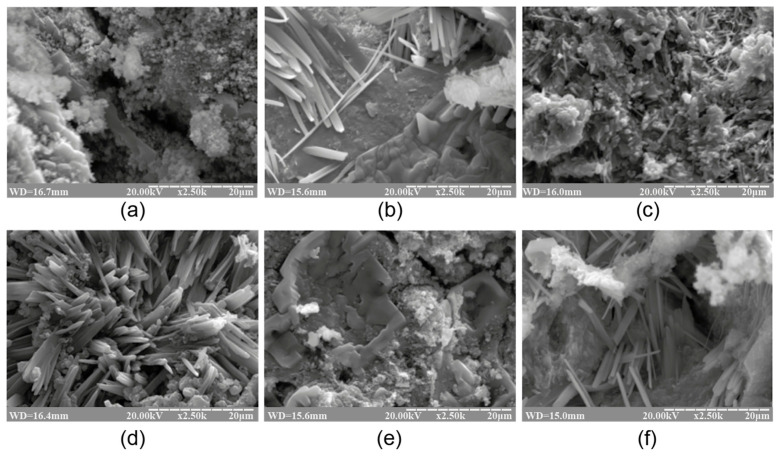
The SEM images of the cleaved surface of the binder with varying K_2_O/(Na_2_O+K_2_O) = 0.15 and SiO_2_/Al_2_O_3_ ratios, namely (**a**) 2, (**b**) 3, (**c**) 4, (**d**) 5, (**e**) 6, and (**f**) 7 (magnification ×2.5K), after curing at t = 80 °C.

Figure 1 and Figure 2 demonstrate the XRD patterns and SEM images of the reaction products represented by the alkaline aluminosilicate hydrates of the hardened binder of the composition with varying ratios of constituent oxides, namely SiO_2_/Al_2_O_3_ = 2…7, K_2_O/(Na_2_O+K_2_O) = 0.15, and (Na_2_O+K_2_O)/Al_2_O_3_ = 1, after curing at t = 80 °C. 

The phase composition of the reaction products of the binders with low ratios (SiO_2_/Al_2_O_3_ = 2…3) was characterized by the following zeolite-like reaction products: analcime (d/n = 0.699; 0.365; 0.336; 0.293 nm), natrolite (d/n = 0.287; 0.243; 0.138 nm), and ussingite (d/n = 0.492; 0.347; 0.295 nm). The amorphous phases presented by the alkaline aluminosilicate hydrates and particles of non-reacted metakaolin were identified (Figure 2).

The hardened binder with SiO_2_/Al_2_O_3_ = 4…5 was characterized by the following zeolite-like reaction products: analcime (d/n = 0.699; 0.365; 0.336; 0.293 nm), sodium heulandite (d/n = 0.509; 0.392; 0.296 nm), potassium heulandite (d/n = 0.342; 0.281; 0.273 nm), and sodium-potassium phillipsite (d/n = 0.498; 0.408; 0.269 nm). The degree of crystallinity of the resulting structure was high as a result of intensive diffraction reflections (Figure 1) and due to the microstructure of the hardened binder (Figure 2).

As mentioned earlier, the modification of the binder with Ca-containing additives resulted in the formation of zeolite-like reaction products of the hybrid type, as follows: calcium–sodium aluminosilicate hydrates and Na- and K-heulandites (the latter products were found in small quantities) [44]. With such a modification, the strength gain of the binder under normal temperatures (t = 20  ±  2 °C) could be reached. The Ca-containing modifying additives used in this study were Portland cement, ground granulated blast furnace slag, Ca(OH)_2_, and CaCO_3_.

#### 3.1.2. Influence of Curing Conditions

**Figure 3:**
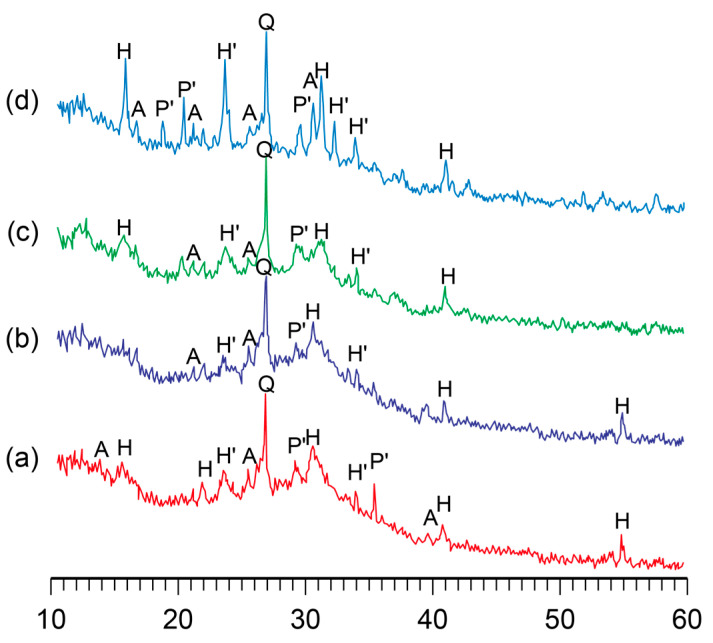
The XRD patterns of the binder with K_2_O/(Na_2_O+K_2_O) = 0.15 and SiO_2_/Al_2_O_3_ = 5 after curing at the following temperatures, °C: (**a**) 20; (**b**) 40; (**c**) 60; (**d**) 80. Abbreviations: Q—quartz; A—analcime; P’—Na–K phillipsite; H—Na heulandite; H’—K heulandite.

**Figure 4:**
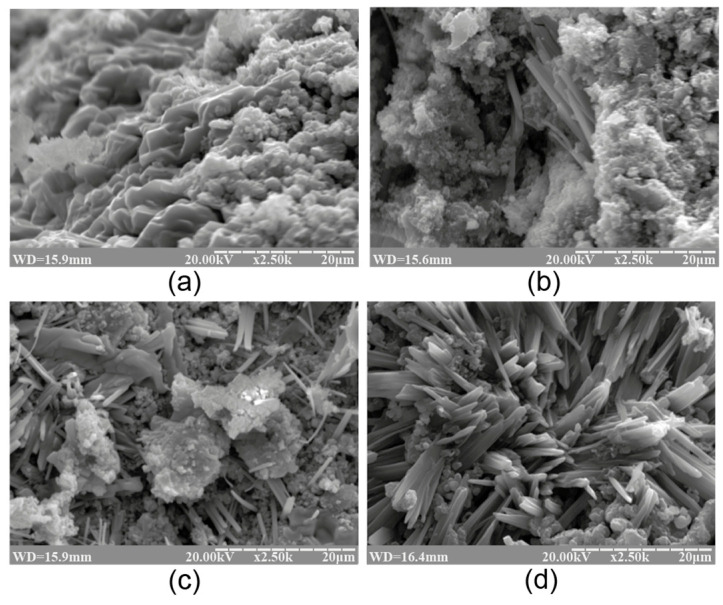
The SEM images of the cleaved surface of the binder with K_2_O/(Na_2_O+K_2_O) = 0.15 and SiO_2_/Al_2_O_3_= 5 after curing at the following temperatures, °C: (**a**) 20; (**b**) 40; (**c**) 60; (**d**) 80 (magnification ×2.50K).

The influence of curing temperature during the hardening of the binder on its phase composition was studied. It was found that the phase composition of the alkaline aluminosilicate hydrate with K_2_O/(Na_2_O+K_2_O)= 0.15 and SiO_2_/Al_2_O_3_ = 5 was characterized by the presence of the following zeolite-like reaction products, analcime, potassium, and sodium heulandite, as well as sodium–potassium phillipsite (Figure 3). With the temperature increase, the structure formation processes in the binder accelerated without affecting the phase composition, resulting in a higher degree of crystallinity of the reaction products (Figure 4).

**Figure 5:**
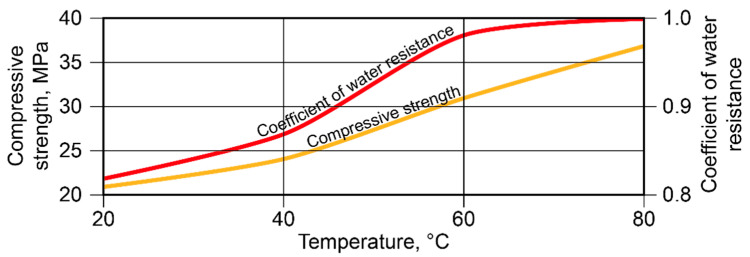
Compressive strength and coefficient of water resistance vs. curing temperature (binder with SiO_2_/Al_2_O_3_ = 5 and K_2_O/(Na_2_O+K_2_O) = 0.15).

With SiO_2_/Al_2_O_3_ = 5 and K_2_O/(Na_2_O+K_2_O) = 0.15, an essential increase in strength took place at t = 40…80 °C. The highest values of the coefficient of water resistance were reached after curing at t = 60…80 °C (Figure 5).

A conclusion was drawn with regard to the binders of the general formula (0.7…1.0Na_2_O+0…0.3K_2_O)∙Al_2_O_3_∙2…7SiO_2_∙nH_2_O that the phase composition of the binder depended mainly on the ratio of oxides and that a curing temperature (in the range of 20…80 °C) accelerated the formation of zeolite-like aluminosilicate hydrates. 

The SiO_2_ /Al_2_O_3_ ratio was found to be the main factor determining the type of reaction products that formed: the higher this ratio, the larger the quantity of silica formed in the crystal lattice in the zeolite-like phases. During the curing of these alkaline aluminosilicate hydrates under normal conditions, allowing them to reach the highest degree of structure crystallinity, the optimal ratio of SiO_2_/Al_2_O_3_ was 4 to 5. The hardened binder with this ratio of oxides was characterized by the highest strength and water resistance.

The addition of potassium ions into the binder composition contributed to the formation of zeolite-like reaction products and an increase in the degree of crystallinity of the phases. To accelerate the formation of alkaline aluminosilicate hydrates under normal conditions, the following quantity of potassium oxide was required: K_2_O/(Na_2_O+K_2_O) = 0.15…0.30. The potassium ions also contributed to the higher water resistance and strength of the hardened binder, regardless of the curing temperature. 

With the rise in temperature from 20 °C to 80 °C, the phase composition of the hardened binder hardly changed. However, this resulted in a higher rate of structure formation and degree of crystallinity.

The microscopic examination of the binders under study showed the presence of a sub-microcrystalline structure with microcrystals ≤3.5 microns in size alongside the amorphous phase. Continuously increasing the adhesion between the sub-microcrystalline and amorphous phases led to the higher strength and better durability of these structures, which, due to their nature, can be exposed to external deteriorating factors without significant resulting damage. The structure of the hardened binder was mainly represented by crystals of hexagonal form, which can be referred to as zeolite-like reaction products such as analcime, zeolite P (with the structure of garronite), and zeolite G (with the structure of chabazite). These reaction products were present in various quantities, depending on the initial binder composition, and differ in their degree of crystallinity (Figure 1, Figure 2, Figure 3 and Figure 4). Changing the ratio of Na_2_O to K_2_O allowed us to obtain the required composition of the reaction products and to keep the structure formation in the binder and its resulting density under control. The density values of the zeolite-like reaction products were as follows: zeolite G (0.205–0.21 nm) < zeolite P (0.230 nm) < analcime (0.224 nm). The maximum density of the binder was obtained through the formation of equal quantities of aluminosilicate hydrates of the analcime and zeolite P types. 

**Figure 6:**
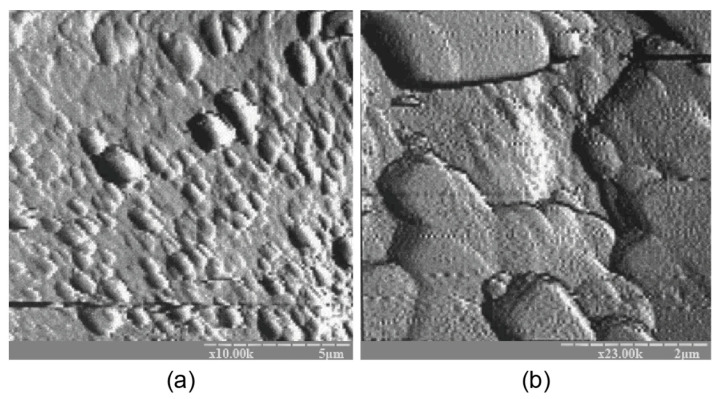
The AFM results for the binder with Na_2_O∙Al_2_O_3_∙4SiO_2_∙10H_2_O modified with the Ca-containing additive and cured for 28 d at t = 20  ±  2 °C: (**a**) magnification ×10 K; (**b**) magnification ×23 K.

The atomic force microscopy (AFM) examination results coincide well with the results of AFM shown in Figure 6. The binder microstructure, prepared with the ratio of H_2_O/Al_2_O_3_ = 12.5, was characterized by the presence of single microcrystals within the range of 0.1 to 0.5 microns that were chaotically distributed in the amorphous phase, testifying to the uneven crystallization process. A conclusion can be drawn about the absence of a clearly expressed stage in the formation of the sub-microcrystalline structure during the transformation of the binder from the amorphous state into the crystalline one. Nucleation of large crystals takes place in the amorphous phase, resulting in significantly slower crystallization and hardening processes. 

**Figure 7:**
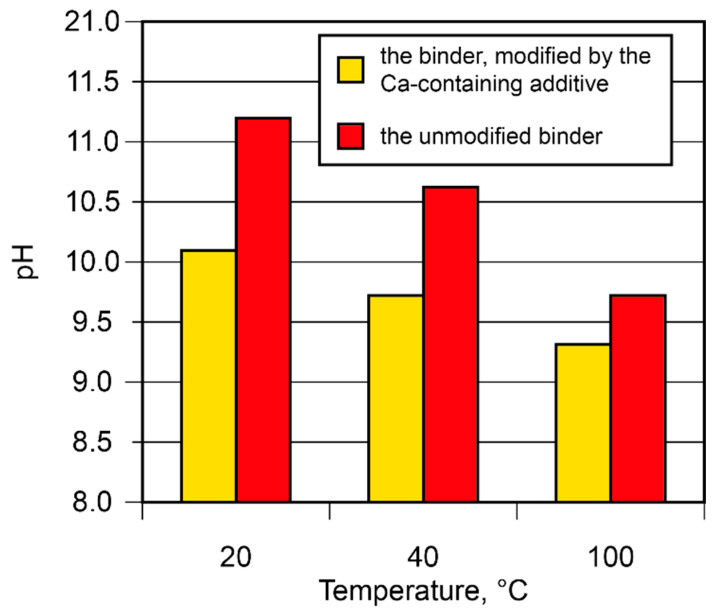
The pH values of the pore fluid vs. binder composition and curing temperature.

The binding degree of the alkali metal ions in the binder was studied. The binding degree depends on the curing conditions and increases as the curing temperature rises (Figure 7). With the rise in temperature from 20 °C to 100 °C, the pH value of the pore fluid tended to decrease (from 11.2 to 9.7). The introduction of the Ca-containing additive reduced the pH value from 10.1 to 9.3. This phenomenon can be attributed to its participation in the binding process, with the formation of such reaction products as amicite (d = 0.564; 0.422; 0.314; 0.272 nm), garronite (d = 0.710; 0.501; 0.410; 0.316; 0.267 nm), and gismondine (d = 0.188; 0.191; 0.273; 0.274; 0.319; 0.324; 0.419; 0.467; 0.494 nm). 

**Figure 8:**
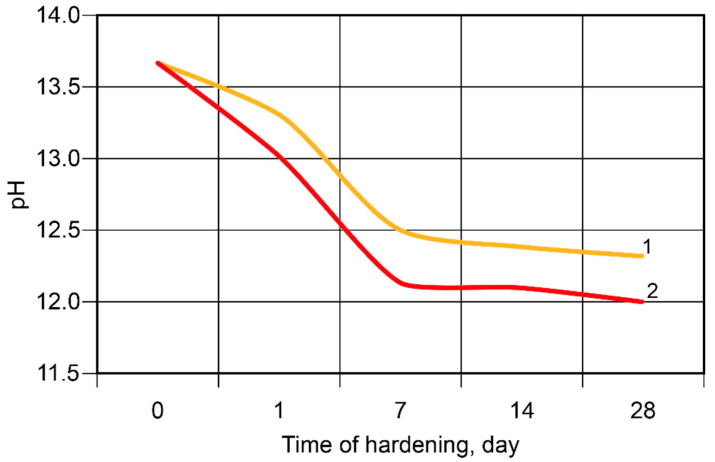
The pH values of the pore fluid vs. time of hardening at t = 20 °C: 1—the binder without modification; 2—the calcium-modified binder.

During the hardening of the binder, the binding process of the alkali metal ions took place, with the most intense changes taking place during the first seven days (Figure 8). The Ca-containing additive accelerated the binding of the ions.

**Figure 9:**
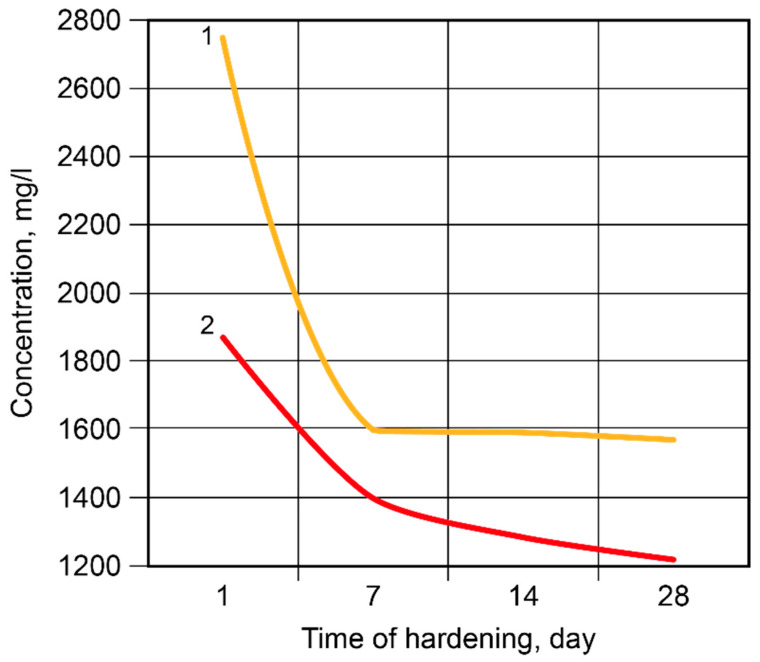
The concentration of the Na^+^-ions in the pore fluid vs. time of hardening: 1—the binder without modification; 2—the Ca-modified binder.

After 2 days, 84.99% of the free Na^+^-ions were bound in the binder matrix without modification and 89.79% were bound in the Ca-modified binder (Figure 9). After 7 days, 91.26% and 92.36% of the ions, respectively, were bound in the binder matrix. Then, the rate of binding became considerably lower, especially in the case of the binder without modification; after 28 days, these values were 8.57% (binder without modification) and 1.20% (Ca-modified binder). The Ca-containing additive accelerated the binding of the Na^+^-ions through the formation of zeolite-like reaction products. 

**Figure 10:**
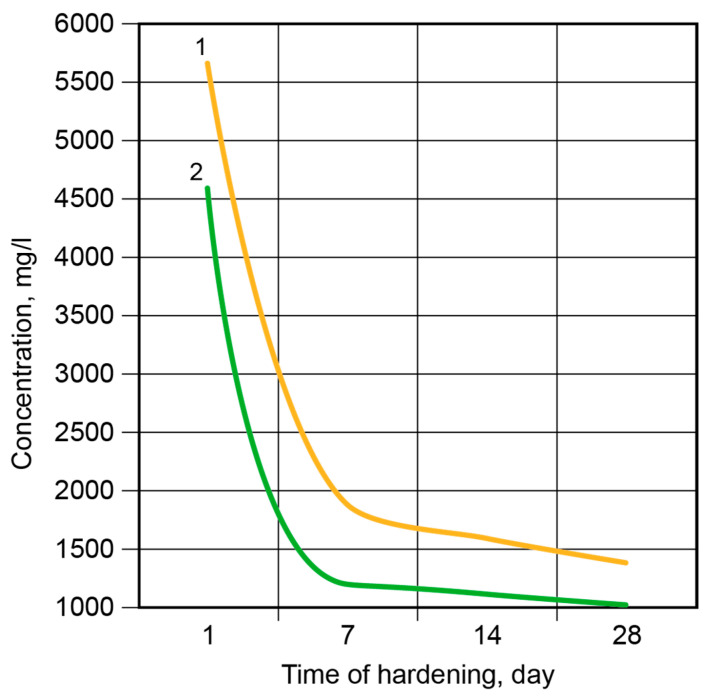
The concentration of K^+^-ions in the pore fluid vs. time of hardening: 1—the binder without modification; 2—the Ca-modified binder.

Figure 10 presents the binding degree of the K^+^-ions in the binder matrix. After 2 days, 95% of the free K^+^-ions were bound in the binder without modification (compared with 96% of the Ca-modified binder).

After 7 days, approximately 99% of the K^+^-ions were bound. Then, the binding rate became considerably lower, and after 28 days, it was 1.25% for the binder without modification and 0.85% for the Ca-modified binder.

### 3.2. Manufacturing Process and Examples of Application of the Alkaline Aluminosilicate Binder

**Figure 11:**
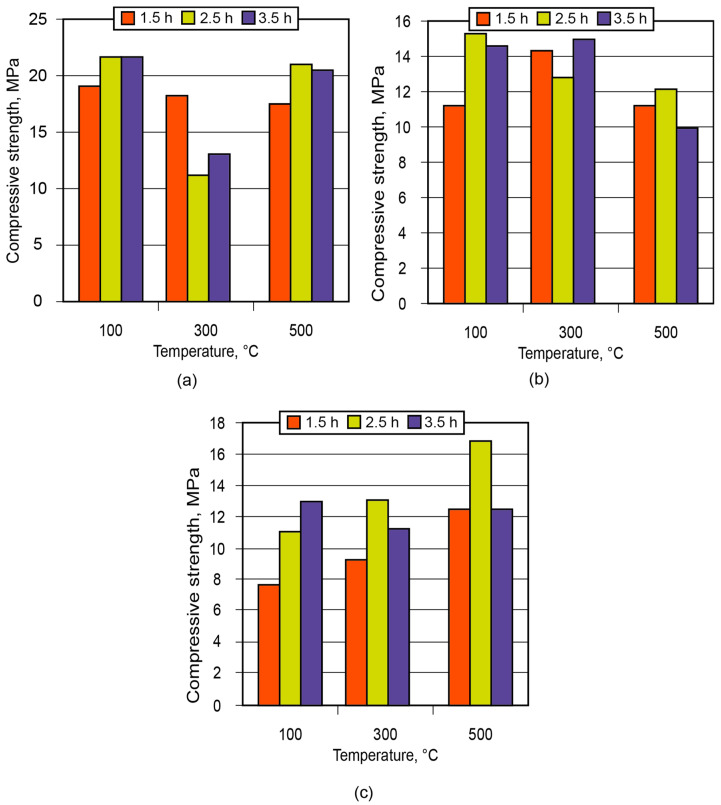
Strength of the ceramic matrix (aluminosilicate component—kaolin) vs. time of conditioning and treatment temperature: (**a**) the binder Na_2_OAl_2_O_3_3.5SiO_2_10.5H_2_O; (**b**) the binder Na_2_OAl_2_O_3_3.5SiO_2_12.5H_2_O; (**c**) the binder Na_2_OAl_2_O_3_3.5SiO_2_14.5H_2_O.

**Figure 12:**
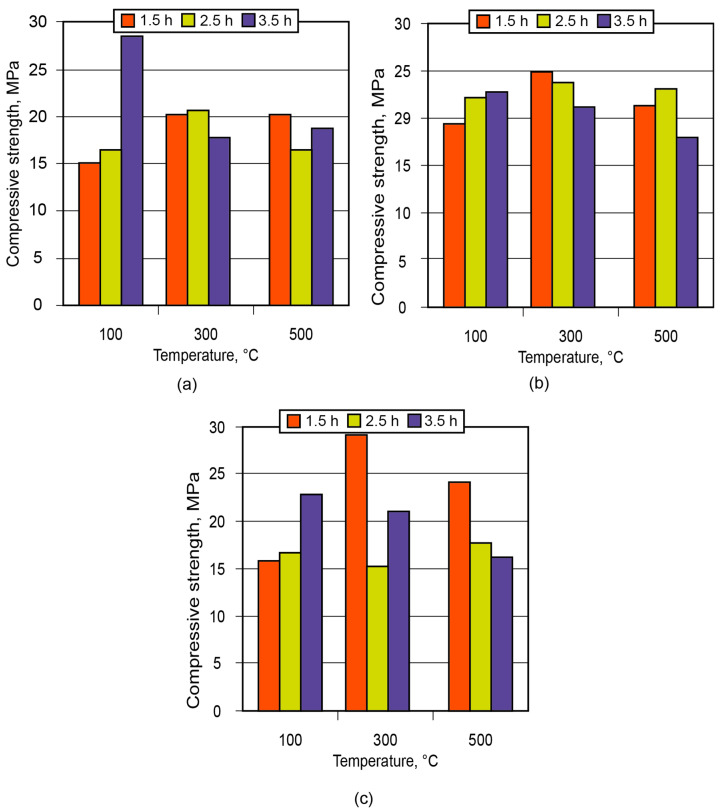
Strength of the ceramic matrix based on metakaolin vs. time of conditioning and treatment temperature: (**a**) binder Na_2_O·Al_2_O_3_·3.5SiO_2_·10.5H_2_O; (**b**) binder Na_2_O·Al_2_O_3_·3.5SiO_2_·12.5H_2_O; (**c**) binder Na_2_O·Al_2_O_3_·3.5SiO_2_·4.5H_2_O.

In order to demonstrate the application of the binder in the manufacture of low-temperature-insulating composite materials, an alkaline aluminosilicate binder of the hydronepheline-analcime type (Na_2_O·Al_2_O_3_·3.5SiO_2_ (10.5; 12.5; 14.5)H_2_O) was chosen as a binding agent. The influence of the number of water molecules contained in the binder produced using kaolin and metakaolin, after conditioning (maturing) of the aluminosilicate blends for 1.5 to 3.5 h and further high-temperature treatment, on the strength of the resulting ceramic matrices (low-temperature ceramics) is shown in Figure 11 and Figure 12.

Analysis of the obtained results showed that the low-temperature ceramic matrix (the alkaline aluminosilicate binder, Na_2_OAl_2_O_3_3.5SiO_2_14.5H_2_O, based on metakaolin) after conditioning for 1.5 h and treatment at t = 300 °C had the highest compressive strength among all the matrices.

**Figure 13:**
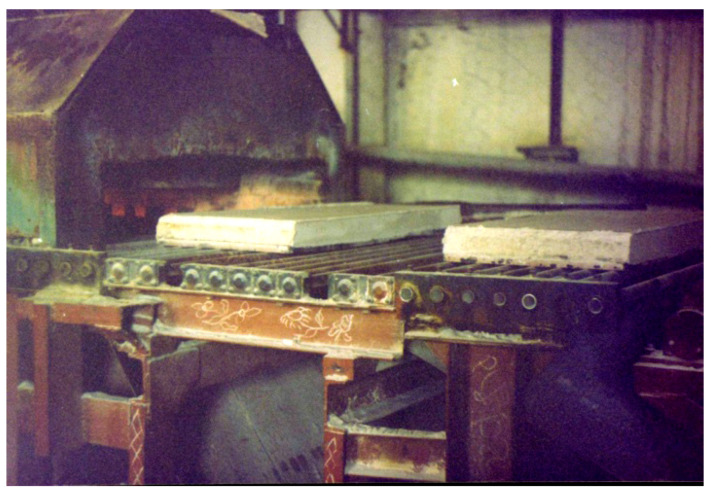
Pilot-scale production of the heat-insulating boards using expanded perlite.

The binder compositions used for the manufacture of perlite-based materials for heat insulation (lightweight concrete) were formulated and tested in pilot-scale production (Figure 13). 

The results of tests of the perlite-based materials are compared in Table 1. The composites prepared using the alkaline aluminosilicate binder were the best for high-temperature insulation applications. 

**Figure 14:**
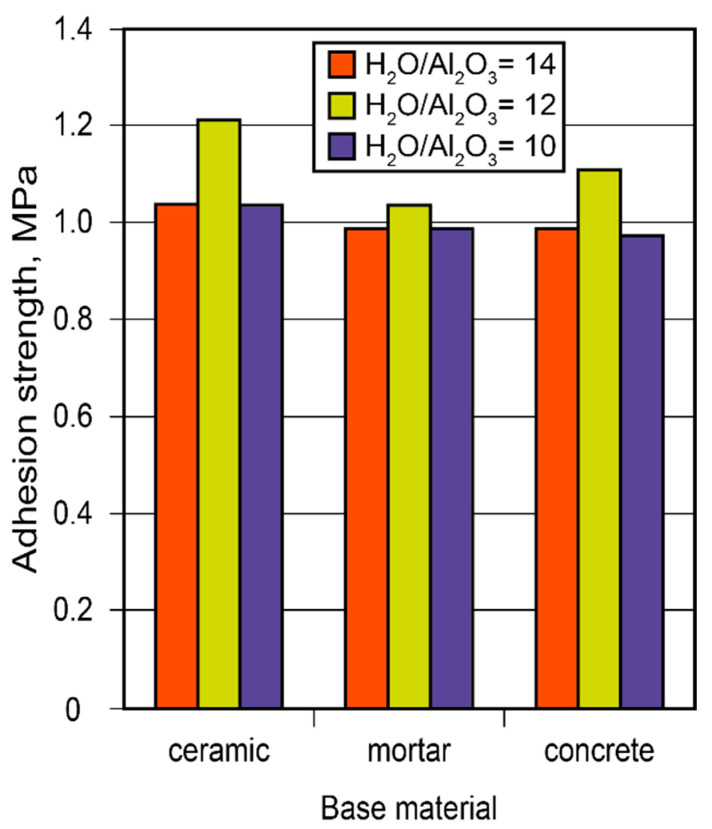
The adhesion strength of the adhesive joints between the adhesive and various other substrates (ceramics, mortar, concrete) vs. binder composition after 28 d.

Considering the constantly increasing restrictions placed on energy consumption, the demand for heat-saving solutions has become a priority. Conserving heat in high-temperature installations is especially critical since the relative cost of energy produced at high temperatures (exceeding 500 °C) is considerably higher than that produced at lower temperatures. Binder formulations for high-temperature heat insulation for the insulation of boilers were developed. The guidelines on how to choose and arrange insulating materials were elaborated based on the study of the adhesion strength of adhesive joints through the design of alkaline-aluminosilicate-binder-based adhesives and using a numerical model for the design of high-temperature heat-insulating materials (Figure 14).

A solution to achieving the heat insulation of industrial boilers was developed. Among the many technical advantages of alkaline aluminosilicate binders as binding agents in the manufacture of adhesives are the following: ecological friendliness, incombustibility, absence of hazardous gases released under exposure to high temperatures, durability, and low cost. These materials are easy to prepare and apply, and they can be produced under almost any condition, in any quantity, with the use of primitive mixing equipment (e.g., electric drill). 

**Figure 15:**
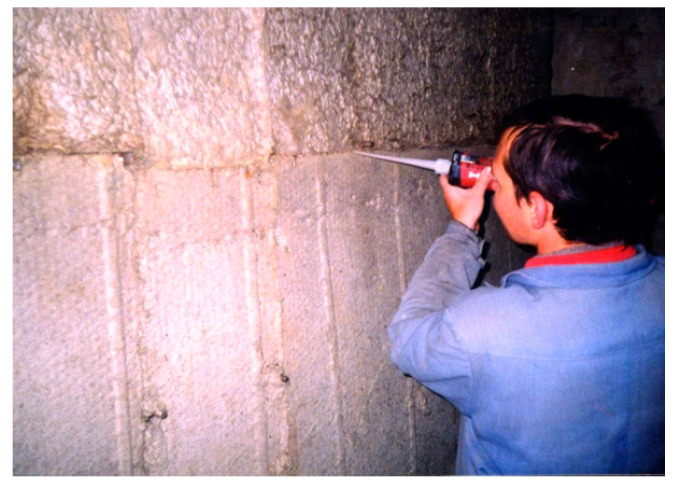
Work on sealing holes in the heat-insulating layer using an injection pistol.

Adhesives prepared using alkaline aluminosilicate binders as binding agents can be used for bonding layers of heat insulation products to each other for the heat insulation of structural elements with geometrically complicated shapes, impregnating basalt fiber cords intended for sealing holes, and for the direct sealing of holes. For these applications, a composition using ground sand as a filler was designed and applied with the help of an injection pistol into holes up to 4 mm in width and with a putty knife for holes ranging from 4 to 20 mm in width (Figure 15). The shelf life of this adhesive composition was between 1 h and 6 h, with a solidification time between 1 h and 8 h, and a strength gain period ranging from 24 h to 48 h, depending on the ambient temperature.

Fire-resistant heat-insulating composite materials were developed from binder-based adhesives and basalt fibers.

**Figure 16:**
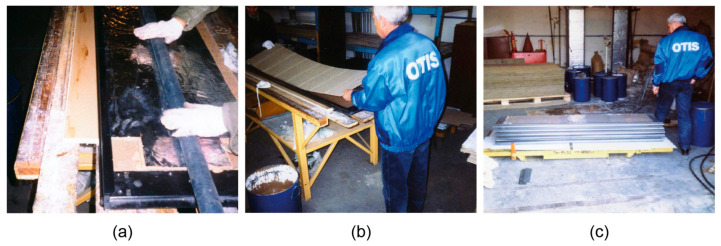
Technological operations: (**a**) adhesive application on aluminum foil; (**b**) gluing of basalt cardboard; (**c**) storage of the ready products (doors).

**Figure 17:**
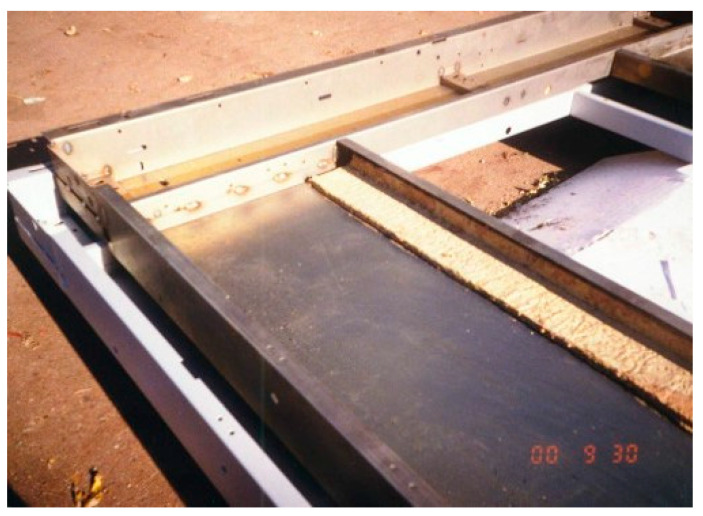
Production of floor frames of the lifts.

One other important problem requiring a solution is how we ensure health and safety in the case of natural disasters and catastrophes in multistoried buildings with many people inside. One of the necessary tasks is to ensure quick localization of the fire source via a fire stop. Lifts and lift shafts should be protected by insulation using mineral materials. A solution to this problem was proposed, involving the use of binder-based adhesives with good fire-stopping ability and good heat insulation properties. These composite materials, due to their inorganic nature, do not release hazardous gaseous substances at high temperatures. A new design for fire-resistant doors was developed, since none of the previously used adhesives meet the requirements for fire-resistant lift shaft doors. Using an alkaline aluminosilicate binder in the manufacturing process of fire-resistant floor frames was also proposed. The manufacture and commercial-scale use of the mentioned products were initiated by the OTIS (Ukraine) (Figure 16 and Figure 17). 

The alkaline-aluminosilicate-binder-based adhesives were tested on their ability to adhere to various materials: a glass crystalline plate, basalt cardboard, and steel and aluminum foil (Table 2).

## 4. Conclusions

1. This study developed an approach to designing an alkaline aluminosilicate binder to be used in a variety of responsible-use applications, for example, for advanced heat- and high-temperature-insulating composite materials with higher efficiency compared with other traditionally used mineral and organic binding agents, including those intended for fire-stopping applications.
2. The structure formation processes in the alkaline aluminosilicate binders were characterized by the following oxide ratios: (Na,K)_2_O/Al_2_O_3_ = 1, SiO_2_/Al_2_O_3_ = 2 to 7, and H_2_O/Al_2_O_3_ = 10 to 15. These caused the formation of zeolite-like reaction products, chiefly composed of aluminosilicate hydrates: analcime, zeolite P, and garronite. The introduction of a calcium-containing additive to the binder composition resulted in the formation of zeolite P and analcime as well as calcium silicate hydrates with the structure of ksonotlite and girolite. The additional quantities of SiO_2_ added to the binder composition determined the predominance of zeolite-like reaction products with an increased content of SiO_2_, i.e., minerals of the Na-shabasite- gmelenite, faujasite, and mordenite types.
3. The hardening process of the binder with H_2_O/Al_2_O_3_ < 10 took place with the formation of aluminosilicate hydrates through the following stages: amorphous, sub-microcrystalline, and crystalline. In the case of the binder with H_2_O/Al_2_O_3_ > 10, the sub-microcrystalline structure was very poorly identified; as a result, the nucleation of large crystals took place in the amorphous phase, leading to slower hardening and crystallization processes and, finally, to lower properties of the resulting cement matrix.
4. Hardening of the binder within 28 days was accompanied through the binding of 93 to 99% of Na^+^- and K^+^-ions in the binder matrix. The introduction of the Ca-containing additive allowed us to accelerate the binding of these ions due to the formation of zeolite-like reaction products of the amicite, garronite, and gismondine types.
5. The effectiveness of the designed binder as a binding agent for use in the manufacture of glues and adhesives is supported by the experience gained from pilot- and small-scale industrial manufacture and use. Such environmentally friendly mineral binder-based composites encompass a variety of insulating materials for heat insulation and fire stop applications.

## Figures and Tables

**Table 1:** Basic physical–mechanical characteristics of the perlite-based materials (binding agents: the alkaline aluminosilicate binder and OPC (reference)).

Characteristic	Density, kg/m^3^			
	150	200	250	300
Alkaline aluminosilicate binder				
Binder content (% by volume)	3.0	6.0	8.0	10.0
Compressive strength (MPa)	0.25	0.30	0.35	0.45
Flexural strength (MPa)	0.15	0.15	0.20	0.30
Heat conductivity at 25 °C (W/(m·°C))	0.062	0.068	0.076	0.082
Ordinary Portland cement (OPC)				
Binder content (kg/m^3^)	−	−	80	110
Compressive strength (MPa)	−	−	0.15	0.25
Flexural strength (MPa)	−	−	0.16	0.20
Heat conductivity at 25 °C (W/(m·°C))	−	−	0.075	0.085

**Table 2:** Characteristics of the adhesion.

Glued Materials	Correlation of the Total Areas at Uniform Tearing, %		
	Cohesive Destruction (in Glue), No More Than	Adhesive Destruction (Contact Zone Material: Glue), No More Than	Destruction in Fibrous Layer, Not Less Than
Plate–cardboard	5	5	90
Cardboard–steel	20	30	50
Plate–steel	20	30	50
Plate–aluminum foil	20	30	50

## Data Availability

The data are not publicly available, as this work was carried out within the framework of the mentioned projects, and its results can be made available on request after registration of a report in the state scientific institution of the Ukrainian Institute of Scientific and Technical Expertise and Information (http://www.uintei.kiev.ua/en) at https://nddkr.ukrintei.ua/.
